# Invasion Fosters Change: Independent Evolutionary Shifts in Reproductive Traits after *Oxalis pes-caprae* L. Introduction

**DOI:** 10.3389/fpls.2016.00874

**Published:** 2016-06-24

**Authors:** Sílvia Castro, Mariana Castro, Victoria Ferrero, Joana Costa, Daniela Tavares, Luis Navarro, João Loureiro

**Affiliations:** ^1^Centre for Functional Ecology and Department of Life Sciences, University of CoimbraCoimbra, Portugal; ^2^Department of Plant Biology, Faculty of Science, University of VigoVigo, Spain; ^3^Department of Ecology and Evolutionary Biology, University of TorontoToronto, ON, Canada

**Keywords:** clonality, evolution of reproduction, Mediterranean regions, pentaploid, polyploidy, reproductive strategy, sexual and asexual reproduction, tristyly

## Abstract

Biological invasions offer optimal scenarios to study evolutionary changes under contemporary timescales. After long-distance dispersal, exotic species have to cope with strong mate limitation, and shifts toward uniparental reproduction have been hypothesized to be selectively advantageous. *Oxalis pes-caprae* is a clonal tristylous species native to South Africa, and invasive in Mediterranean regions worldwide. It reproduces sexually and asexually but the importance of each strategy differs between ranges. Native populations reproduce mostly sexually while in invasive ones asexual reproduction is the prevailing strategy due to the dominance of pentaploid monomorphic populations. Nevertheless, two contrasting scenarios have been observed after introduction: transition toward clonality, and re-acquisition of sexuality fueled by multiple introductions of compatible mates. Here, we aimed to assess evolutionary changes of reproductive traits in *O. pes-caprae* invasive populations and evaluate whether these traits could be related with invasion success and prevalence of certain forms in the western Mediterranean basin. Sexual and asexual reproduction traits were quantified under optimal conditions in a common garden experiment including native and invasive sexual, predominately asexual, and obligated asexual individuals. Different reproductive, ecological, and genetic constraints created by long-distance dispersal seem to have generated different selective pressures in sexual and asexual traits, with our results supporting evolutionary changes in invasive populations of *O. pes-caprae*. Native plants had higher sexual fitness, while a transition toward clonality was clear for invasive forms, supporting clonal reproduction as a major trait driving invasion. Differences were also observed among invasive plants, with sexual forms having increased dispersal potential; thus, they are expected to be in advantage in comparison with predominantly asexual and obligated asexual plants, and may become widespread in the future. Historical processes, like the initial introduction of predominantly asexual forms followed by sexual forms more recently, could be in the origin of current distribution patterns of *O. pes-caprae* in the western Mediterranean. This study shows that invasion processes are very dynamic and that ecological and genetic constraints determined by the invasion process may originate different reproductive strategies that are likely to determine invasion success.

## Introduction

Biological invasions are a serious threat to biodiversity and have long been recognized to comprise significant ecological and evolutionary consequences, not only for the communities being invaded, but also for the invasive species themselves (Brown and Eckert, 2005; Barrett et al., 2008; Pyšek et al., 2012; Oduor, 2013). For these reasons, since the seminal works of Elton (1958) and Baker and Stebbins (1965), biological invasions have attracted much attention of researchers in an attempt to identify traits that might confer an advantage during colonization of new habitats (Pyšek and Richardson, 2007; Hayes and Barry, 2008; van Kleunen et al., 2010) and to understand the biotic and abiotic factors that determine invasion success (e.g., Souza et al., 2011; Wisz et al., 2013). It also became clear that the introduction of sub-populations in new ecological scenarios generates valuable oportunities to study evolutionary transitions over contemporary time scales (e.g., Sakai et al., 2001; Brown and Eckert, 2005; Barrett et al., 2008; Prentis et al., 2008). These studies contribute to a better understanding of the factors triggering a successful invasion, and provide new insights on the evolutionary history of specific traits, such as those related to the reproductive system (Barrett et al., 2008; Barrett, 2011).

Reproduction is one of the key factors involved in the successful establishment and spread of a given organism after long-distance dispersal (e.g., Sakai et al., 2001; Barrett et al., 2008; Hayes and Barry, 2008; Castro-Díez et al., 2014; Moravcová et al., 2015). Reproductive modes determine the production, dispersal and genetic composition of propagules, thus influencing the genetic and demographic structure of populations, as well as the dispersal ability and evolutionary potential of introduced individuals or sub-populations, that, by its turn, will also determine the reproductive strategy (Sakai et al., 2001; Novak and Mack, 2005; Barrett et al., 2008; Ness et al., 2010). Flowering plants exhibit an outstanding diversity of reproductive strategies, from sexual to asexual modes and from self-compatible to obligated outcrossers (reviewed in Barrett, 2002), that frequently occur in combination and reveal liability under certain ecological and genetic stressful conditions (Dorken and Eckert, 2001; Eckert, 2002; Goodwillie et al., 2005; Herben et al., 2015). Thus, the relative contribution of each strategy to the fitness of a population/individual is expected to vary under the novel conditions and will play a major role in the establishment and spread of the introduced individual(s) (e.g., Brown and Eckert, 2005; Lui et al., 2005; Barrett et al., 2008; Silvertown, 2008). Sexual reproduction provides the possibility for increasing genetic diversity through recombination, thus contributing not only to ameliorate loss of genetic diversity due to founder events, but also to fuel the opportunities for local adaptation and the ability of colonizers to respond to unpredictable environmental fluctuations in the new range(s) (Eckert, 2002; Novak and Mack, 2005; reviewed in Barrett, 2011). Despite the clear advantages of sexuality, asexual reproduction might be favored under unreliable circumstances, as it provides reproductive assurance and enables the persistence of individuals in unfavorable habitats for sexual reproduction or avoids the costs associated with sexual reproduction, allowing small populations and adaptive genotypes to rapidly establish and spread (Eckert, 2002; Barrett, 2015; reviewed in Vallejo-Marín et al., 2010).

Long-distance dispersal is frequently associated with strong founder effects and loss of genetic diversity, thus exposing founder individual(s) to strong mate limitation both at the establishment of the first viable population(s) and during range expansion (Baker, 1955, 1965; Stebbins, 1957). This is particularly relevant in obligated outcrossing species, such as self-incompatible or heterostylous plants, in which compatible mates might be lost during long-distance dispersal (e.g., Ornduff, 1987; Hollingsworth and Bailey, 2000; Barrett et al., 2008; Zhang et al., 2010). Under this scenario, a switch to uniparental reproduction, either through self-fertilization or increased asexual reproduction, might be selectively advantageous and foster invasion (reviewed in Pannell et al., 2015). Transitions to asexual reproduction or selfing have been documented for several introduced species, such as the clonals *Fallopia japonica* in the UK (Hollingsworth and Bailey, 2000), *Eichhornia crassipes* in China (Zhang et al., 2010), *Oxalis pes-caprae* in Mediterranean regions (Baker, 1965; Ornduff, 1987), and *Arundo donax* in Australia (Haddadchi et al., 2013), and the self-compatibles *Echinochloa microstachya* in Australia (Barrett and Husband, 1990), *Echium plantagineum* in Australia and Canary Islands (Petanidou et al., 2012), and *Gomphocarpus physocarpus* in Australia (Ward et al., 2012). Additionally, higher rates of uniparental reproduction in introduced and in invasive species compared with natives or with species that failed to establish, have been reported by several studies (Mulligan and Findlay, 1970; Rambuda and Johnson, 2004; Silvertown, 2008; van Kleunen et al., 2008; Marco et al., 2010). Nevertheless, comparative studies of plant reproductive strategies in native and invaded ranges are scarce (but see Brown and Eckert, 2005; Lavergne and Molofsky, 2007; Petanidou et al., 2012).

Sexual and asexual reproductive strategies frequently co-occur in flowering plants and, although this dual strategy was proven to be advantageous (Silander, 1985; Bengtsson and Ceplitis, 2000; Van Drunen et al., 2015), it can also lead to allocation trade-offs and antagonist interactions between reproductive modes, such as the interference generated by clonal growth in the opportunities for mating (Handel, 1985; Vallejo-Marín et al., 2010; Barrett, 2015; Van Drunen et al., 2015). By reducing the number of mating partners and by increasing the opportunities for geitonogamous pollen dispersal, clonal growth interferes with sexual reproduction in reducing not only the offspring sired, but also its quality and fitness (e.g., Handel, 1985; Charpentier, 2002; Somme et al., 2014; but see Van Drunen et al., 2015). Allocation trade-offs occur when the production of sexual and asexual structures compete for the resources available from the total resource pool (van Kleunen et al., 2002; Thompson and Eckert, 2004; Liu et al., 2009), or through the replacement of sexual structures by asexual ones or vice-versa (e.g., production of inflorescences instead of vegetative shoots, Geber et al., 1992; production of bulbils in the inflorescences instead of flowers, Ronsheim and Bever, 2000; or production of flowers from meristems that in previous years resulted in vegetative tissue, Savinykh, 2003). Therefore, it is expected that differential fitness of the two strategies will affect the balance between sexual and asexual reproduction in the population over time (Silvertown, 2008; Vallejo-Marín et al., 2010; Van Drunen et al., 2015). If strong trade-offs between investment in sexual and asexual reproduction occur, rapid clonal expansion may limit allocation to flowering and seed production (Vallejo-Marín et al., 2010). However, evidence for fitness trade-offs between sexual and asexual reproduction is ambiguous (Van Drunen et al., 2015). Although several studies support a trade-off between the two strategies (e.g., van Kleunen et al., 2002; Thompson and Eckert, 2004; Liu et al., 2009; Van Drunen and Dorken, 2012), studies at the genet level are scarce and many of them failed to detect such trade-offs between reproductive strategies (Vallejo-Marín et al., 2010; Van Drunen and Dorken, 2012).

*Oxalis pes-caprae* is a clonal tristylous species native to South Africa and invasive throughout all Mediterranean regions of the world. This species reproduces by two contrasting strategies: asexually through the profuse production of bulbs (Pütz, 1994; Vilà et al., 2006) and sexually trough a highly specialized mechanism, tristyly and heteromorphic self-incompatibility system (Ornduff, 1987) that promote cross-fertilization and increased genetic diversity (Barrett, 2002). Sexual and asexual reproduction occurs in both native and invaded areas, but the contribution of each reproductive mode differs between ranges (Castro et al., 2007, 2013; Ferrero et al., 2015). In the native range, isoplethic populations occur (Ornduff, 1987; Turketti, 2010; Ferrero et al., 2015), i.e., populations with similar proportions of the three reciprocal style morphs (long-, mid-, and short-styled morphs, hereafter L-, M-, and S-morph, respectively), indicating that populations are in equilibrium and that sexual reproduction is expected to be the main reproductive mode. In the invaded range, two different scenarios appear to be occurring. Until very recently, the main scenario was a transition toward clonality in which the pentaploid (5*x*) S-morph was the dominant form, and thus asexual reproduction through bulbs has been pointed as the prevailing mechanism of reproduction and spread (Baker, 1965; Ornduff, 1987; Castro et al., 2007). Additionally, a complete sterile double-flowered form was also reported to be successfully spreading in south western Iberian Peninsula (Castro et al., 2007). However, we have recently detected the re-acquisition of sexual reproduction likely fueled by multiple introductions of compatible mating partners [tetraploid (4*x*) L-, M-, and S-morph individuals; Castro et al., 2013; Ferrero et al., 2015]. This is the first study exploring the role of reproductive traits in the invasion success of *O. pes-caprae*.

The objective of this study was to quantify changes in reproductive traits in invasive populations of *O. pes-caprae* and evaluate whether these differences could explain the prevalence of some floral forms in the invaded range of the western Mediterranean basin and be involved in the invasion success of this species. We compared the investment in sexual and asexual reproduction between native and invasive individuals, and among sexual (4*x* L-, M-, and S-morph), predominately asexual (5*x* S-morph) and obligated asexual individuals (4*x* sterile double-flowered form) found in the invaded range. Based on the invasion history of *O. pes-caprae* and on a trade-off hypothesis between allocation to sexual and asexual reproduction, we expected that, in the invaded range, selection has promoted individuals with an increased capacity for investment in asexual reproduction in detriment of sexual reproduction, especially among the mostly clonal forms; in sexual forms the trade-off between the two strategies might be more dependent of the environmental context. Still, the low sexual success of sexual forms in the invaded area (due to low mate availability and/or genetic depauperated populations; Castro et al., 2013; Ferrero et al., 2015) may generate a context promoting asexuality in comparison with sexual forms from native populations. Thus, we hypothesized that asexual forms would have significantly higher asexual potential than sexual forms in order to become dominant in the invaded range, and that both would have significantly higher asexual potential than sexual forms from the native area where sexual reproduction prevails. Our findings are discussed in the light of biological invasions and of the role of reproductive traits in successful invasion.

## Materials and Methods

### Plant Species

*Oxalis pes-caprae* L. (Oxalidaceae), Bermuda buttercup, is a geophyte with a deeply buried annual bulb that produces subterranean stems bearing a rosette of leaves and several inflorescences of yellow flowers arranged in umbellate cymes (Vilà et al., 2006; Sánchez-Pedraja, 2015). It is a tristylous species with a heteromorphic self-incompatibility system (Ornduff, 1987). Thus, the production of viable offspring only occurs after legitimate pollination between individuals with reciprocal style morphs. Double-flowered sterile individuals have also been frequently observed in the western Mediterranean basin (Castro et al., 2007) and sporadically in South Africa (Salter, 1944; Suda and Oberlander, personal communication). The Bermuda buttercup has a high capacity for asexual reproduction through a profuse production of bulbs. The main bulb produces a fasciculate root with contractile properties that grows deeper in the soil some centimeters each year (Pütz, 1994), and later in the season or under stressful conditions (e.g., soil perturbation), the subterranean stems produce a high number of small bulbs (Young, 1968; Verdaguer et al., 2010; authors personal observations). Furthermore, *O. pes-caprae* is a polyploid species, with diploid (2*n* = 2*x* = 14 chromosomes), tetraploid (2*n* = 4*x* = 28 chromosomes), and pentaploid (2*n* = 5*x* = 35 chromosomes) individuals. In South Africa, all cytotypes have been reported, although the 5*x* cytotype appears to be extremely rare (Ornduff, 1987; te Beest et al., 2012; Ferrero et al., 2015). Contrarily, the 5*x* is the dominant cytotype in the invaded ranges worldwide, although in Australia and recently in the western Mediterranean region, the 4*x* has also been reported (Symon, 1961; Michael, 1964; Castro et al., 2007, 2013).

The Bermuda buttercup was introduced into the Mediterranean basin in the end of the 18th century, most probably multiple times (Vignoli, 1937; Galil, 1968; Signorini et al., 2011), and spread widely afterward. The species was soon recognized as a weed in several Mediterranean areas (e.g., Sicily, Hildebrand, 1887; Canary Islands, Morris, 1895; Algeria, Ducellier, 1914; Balearic Islands, Knoche, 1922; Malta and neighbor islands, Borg, 1927; Tunisia, Chabrolin, 1934), including Portugal where it was described as abundant in orchards (Henriques, 1920; Vasconcelos and Moreira, 1976). Early introductions of the plant occurred due to its ornamental value, and later through soil movement in agriculture, horticulture, and gardening (Michael, 1964; Signorini et al., 2011). Still, the routes of (repeated) introduction to the Mediterranean basin and other invaded regions are not clear.

### Field Sampling

Extensive field sampling for bulb harvesting was conducted during February and March 2010 in the invaded range of the western Mediterranean basin (MB), and during August 2011 in the native area, South Africa (SA). All the necessary permits for plant collection were obtained. In the invaded range, an additional effort was made to sample throughout the regions where trimorphic populations and the sterile double-flowered form are more common (Castro et al., 2007, 2013). All floral forms and cytotypes found in this invaded range were included in our study, i.e., the 4*x* L-morph, 4*x* M-morph, 4*x* S-morph, 5*x* S-morph, and the 4*x* sterile double-flowered individuals (Supplementary Table 1). In the native range, the field sampling was conducted across most of the latitudinal and longitudinal distribution of the species (Salter, 1944; Supplementary Table 1). Our extensive sampling in the native range confirmed previous results showing that the 5*x* S-morph and the double-flowered individuals are extremely rare in South Africa (Ferrero et al., 2015). For this reason, only 4*x* L-morph, 4*x* M-morph, and 4*x* S-morph native plants were included in this comparative study. In each population, we sampled bulbs from 10 individuals per floral form, separated at least 5-m apart to avoid re-sampling clones of the same individual. Sampled populations were characterized for style morph frequency and cytotype composition as described in Castro et al. (2013).

### Common Garden Experiment

To investigate if there were differences in sexual and asexual reproductive traits between native and invasive plants, we conducted a common garden experiment at the Botanical Garden of the University of Coimbra, where individual plants from both areas were grown outdoors under similar optimal conditions. To remove potential maternal effects, bulbs from SA and the MB were grown for one and two generations, respectively, before sexual and asexual investment traits were measured. In June 2012, all bulbs were harvested and stored in paper bags. During September 2012, they were weighed and weight values were recorded as initial bulb weight. The analysis of the dispersion of the initial bulb weight allowed us to select one bulb per individual matching similar overall mean weights (mean ± SD, 0.463 ± 0.086 g). In total, 338 bulbs were selected, representing 29 populations and 137 individuals from the native area, and 13 populations and 201 individuals from the invaded range (Supplementary Table 1). This selection reflected the different reproductive strategies found in SA and MB: sexual (4*x* L-, M-, and S-morphs), predominately asexual (5*x* S-morph), and obligated asexual (4*x* sterile double-flowered form).

Bulbs were individually planted ∼2.0 cm below the soil surface in 2 L plastic pots (9.6 cm × 9.6 cm × 21.5 cm) filled with commercial substrate, and pots were randomized at the beginning of the experiment. Before flowering, plants were covered with a mosquito net to avoid undesirable pollination. To characterize sexual and asexual reproduction performance, we measured the following traits: (a) bulb viability, (b) occurrence of flowering; (c) floral display; (d) biomass invested in sexual and asexual structures; and (e) production of diaspores through sexual and asexual means (fruit, seed, and bulb production). During the flowering peak, we classified each individual as either vegetative or reproductive, and we collected one flower per inflorescence, when produced, into individual paper bags for later estimation of: (a) mean flower weight and (b) total weight investment in flowers per plant. Inflorescences were periodically monitored, and were collected when senescent, allowing us to simultaneously assess: (a) the total number of flowers produced per plant, i.e., floral display, and (b) the total investment in inflorescences measured as dry weight. Total and average investments in the production of sexual structures were estimated for each plant. Fruit and seed production were obtained by cross pollinating three flowers per plant using reciprocal style morphs of the same area of origin. Fruit set was calculated as the proportion of flowers that developed into fruits and seed production as the mean number of seeds per fruit. We calculated a measure of sexual potential for each plant by multiplying the total number of flowers produced by the mean fruit and seed production. Bulbs were harvested by the end of the season. The investment in asexual structures was quantified by assessing the number of bulbs produced per plant, and total and mean bulb weight per plant.

### Statistical Analysis

Data was grouped according to the following criteria: (a) area of origin (South Africa native range, SA, and invaded range of the Mediterranean Basin, MB) and (b) reproductive strategy. We defined reproductive strategy as: *sexual*, including 4*x* plants with morphologically functional flowers (Sex); *predominantly asexual*, 5*x* S-morph plants that reproduce mostly asexually despite some sporadic ability to produce viable offspring (Asex; Castro et al., 2013; Costa et al., 2014, in press); and, *obligated asexual*, 4*x* double-flowered sterile mutants (St). Accordingly, all individuals were assigned to one of the following groups: South African 4*x* sexual forms (SA4*x*Sex), Mediterranean basin 4*x* sexual forms (MB4*x*Sex), Mediterranean basin 5*x* predominantly asexual form (MB5*x*Asex) and Mediterranean basin 4*x* obligated asexual form (MB4*x*St, double-flowered sterile mutant).

The above groups were defined as fixed factor in generalized linear mixed models (GLMM) to assess differences in sexual and asexual traits. GLMMs enabled us to model variables that did not completely fulfill the assumptions of a standard linear model and had the advantage to allow the incorporation of random factors in the models (Bolker et al., 2009). Although the initial bulb weight was fairly homogenous, this variable was included as covariate to account for possible differences caused by bulb weight. Population and individual were defined as random factors and these were removed from the models whenever their variance was lower than the variance of the residuals (Bolker et al., 2009). When both random factors were removed, a generalized linear model (GLM) was used instead (Supplementary Table 2). A binomial distribution with a logit link function was used to model bulb viability and probability of flowering; a Poisson distribution with a log link function was used to model the number of flowers, inflorescences and bulbs per plant; and a Gaussian distribution with an identity link function was used to model the mean number of flowers per inflorescence, mean flower and inflorescence weight, total flower and inflorescence weight per plant, total weight of sexual structures per plant, fruit set (arcsine transformed), mean seed production, sexual potential, mean and total bulb weight. In all cases, differences between least-square means were tested pairwise through multiple comparisons. To evaluate the existence of trade-offs between sexual and asexual investments, correlations between the amount of biomass invested in sexual and asexual structures were calculated for the entire dataset and for each group separately. All statistical analyses were performed in R version 3.1.1 (R Core Team, 2014) using the packages “car” for GLMs and Type-III analysis of variance (Fox and Weisberg, 2015), “nlme” for linear and non-linear mixed models (Pinheiro et al., 2015), and “multcomp” for multiple comparisons after Type-III analysis of variance (Hothorn et al., 2008), and “stats” for GLMs (R Core Team, 2014).

## Results

### Bulb Viability and Probability of Flowering

Results from all statistical analyses are summarized in Supplementary Table 2. Bulb viability was high, varying between 88% in invasive sexual individuals (MB4*x*Sex) and 94% in native sexual individuals (SA4*x*Sex), with no statistically significant differences being observed among groups (χ^2^_3,338_ = 3.49, *P* = 0.322; Supplementary Figure 1A).

The probability of producing floral structures differed among groups (χ^2^_3,305_ = 11.23, *P* = 0.011), with the obligated asexual individuals (MB4*x*St) having a significantly higher probability to remain vegetative than the other invasive groups (i.e., MB4*x*Sex and MB5*x*Asex; *P <* 0.05), while native sexual plants had intermediate values between the two extremes (Supplementary Figure 1B).

### Sexual Traits: Floral Display

**FIGURE 1:**
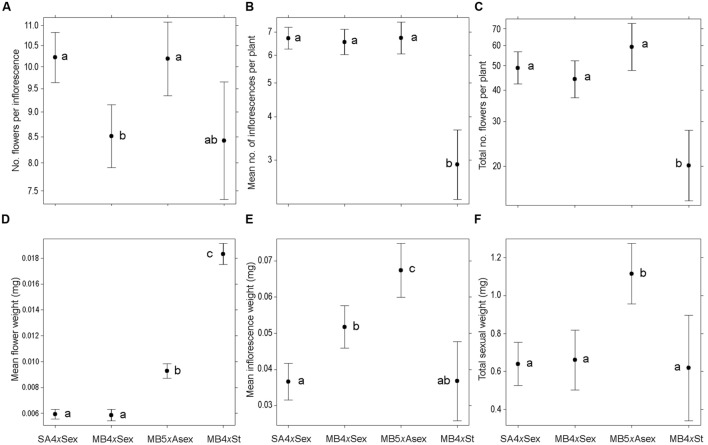
**Floral display and biomass invested in sexual reproductive structures among different forms of *Oxalis pes-caprae* from its native and invaded areas and with distinct reproductive strategies: South African 4*x* sexual forms (SA4*x*Sex), Mediterranean basin 4*x* sexual forms (MB4*x*Sex), Mediterranean basin 5*x* predominantly asexual form (MB5*x*Asex), and Mediterranean basin 4*x* obligated asexual form (MB4*x*St, sterile double-flowered form). (A)** Mean number of flowers per inflorescence; **(B)** Mean number of inflorescences per plant; **(C)** Total number of flowers per plant; **(D)** Mean flower weight (mg); **(E)** Mean inflorescence weight (mg); **(F)** Total weight of sexual structures per plant (mg). Values are given as model-adjusted back-transformed least-square means and 95% confident intervals. Significant differences among factors are indicated with different letters (*P* < 0.05).

Floral display differed among groups in terms of the number of flowers per inflorescence (χ^2^_3,276_ = 19.07, *P <* 0.001), number of inflorescences per plant (χ^2^_3,276_ = 47.89, *P <* 0.001) and total number of flowers per plant (χ^2^_3,276_ = 31.68, *P <* 0.001; **Figures 1A–C**). The native sexual plants and the invasive predominantly asexual plants (MB5*x*Asex) produced inflorescences with more flowers than the invasive sexual individuals (MB4*x*Sex; *P <* 0.05), while the obligated asexual form (MB4*x*St) had a lower and highly variable mean value not differing from the other three groups (**Figure 1A**). The groups with ability for sexual reproduction (SA4*x*Sex and MB4*x*Sex), even if only sporadically (MB5*x*Asex), produced significantly more inflorescences than the obligated asexual individuals, resulting in larger total floral display per plant (*P <* 0.05; **Figures 1B,C**).

Significant differences among groups were also observed in the biomass invested for the production of sexual structures (**Figures 1D–F**, Supplementary Figure 1), namely in the mean flower and inflorescence weight (χ^2^_3,276_ = 820.80, *P <* 0.001 and χ^2^_3,276_ = 51.78, *P <* 0.001, respectively; **Figures 1D–E**), total flower and inflorescence weight per plant (χ^2^_3,276_ = 28.08, *P <* 0.001 and χ^2^_3,276_ = 44.39, *P <* 0.001, respectively; Supplementary Figures 1C,D), and total weight of sexual structures per plant (χ^2^_3,276_ = 32.30, *P <* 0.001; **Figure 1F**). Obligated asexual individuals (MB4*x*St) had significantly heavier flowers, followed by the MB5*x*Asex, and the native and invasive sexual individuals had lower flower weights (*P <* 0.05; **Figure 1D**). The same trend was observed for total flower weight per plant, except for the obligated asexual individuals (MB4*x*St) which produced less inflorescences (**Figure 1B**) and consequently less flowers (**Figure 1C**), lower total flower weight (**Figure 1F**) and reduced investment in total flower biomass (Supplementary Figure 1C). A different scenario was found for mean inflorescence weight, which was significantly lower for the obligated asexual individuals and sexual native plants. Sexual invasive individuals presented intermediate inflorescence weight, and invasive predominantly asexual plants had significantly heavier inflorescences (*P <* 0.05; **Figure 1E**; a similar pattern is observed for the total inflorescence weight per plant; Supplementary Figure 1D). Despite the differences in the number and biomass of reproductive structures among all groups, the total investments in the production of sexual structures per plant did not differ among groups except for the MB5*x*Asex, which presented significantly higher weights (*P <* 0.05; **Figure 1F**).

### Production of Dispersal Units: Sexual and Asexual Strategies

**FIGURE 2:**
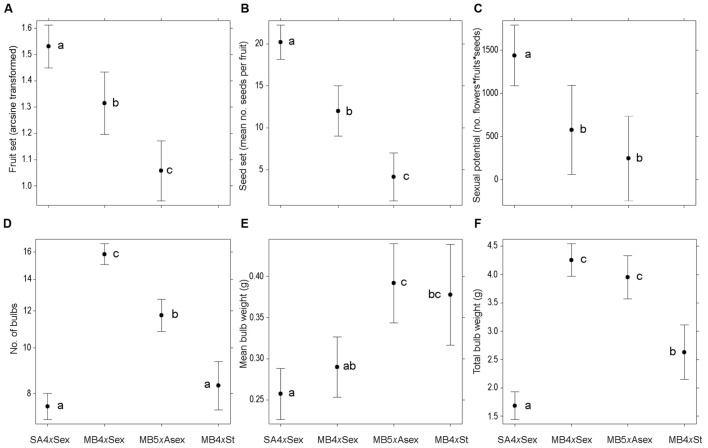
**Production of sexual and asexual diaspores among different forms of *Oxalis pes-caprae* from its native and invaded areas and with distinct reproductive strategies: South African 4*x* sexual forms (SA4*x*Sex), Mediterranean basin 4*x* sexual forms (MB4*x*Sex), Mediterranean basin 5*x* predominantly asexual form (MB5*x*Asex) and Mediterranean basin 4*x* obligated asexual form (MB4*x*St, sterile double-flowered form). (A)** Fruit set, given as arcsine of the proportion of flowers developing into fruit; **(B)** Seed production, given as the mean number of seeds per fruit; **(C)** Sexual potential, given as a measure of sexual ability of the plant obtained multiplying the number of flower produced in a plant by the fruit and seed production; **(D)** Mean number of bulbs per plant; **(E)** Mean bulb weight (mg); **(F)** Total weight of the bulbs produced per plant (mg). Values are given as model-adjusted back-transformed least-square means and 95% confident intervals. Significant differences among factors are indicated with different letters (*P <* 0.05).

Sexual fitness differed significantly among groups (fruit set: χ^2^_2,251_ = 47.38, *P <* 0.001; seed production: χ^2^_2,246_ = 89.44, *P <* 0.001; and sexual potential: χ^2^_2,251_ = 15.77, *P <* 0.001). Fruit and seed production were significantly lower in invasive plants than in native ones and, among invasive groups, it was significantly lower in the predominantly asexual individuals (*P <* 0.05; **Figures 2A,B**). The calculation of a measure of sexual potential revealed that native sexual individuals had a significantly higher success than invasive plants (*P <* 0.05); however, no significant differences were detected in sexual potential between sexual invasive and predominantly asexual invasive individuals (**Figure 2C**).

Asexual traits also differed significantly among groups, either measured as number of bulbs (χ^2^_3,305_ = 350.13, *P <* 0.001), mean bulb weight (χ^2^_3,305_ = 27.36, *P <* 0.001) or total bulb weight per plant (χ^2^_3,305_ = 211.13, *P <* 0.001). Invasive sexual plants produced more bulbs per plant than predominantly asexual individuals, which also produced more bulbs than native sexual and invasive obligated asexual individuals (*P* < 0.05; **Figure 2D**). However, predominantly asexual individuals had significantly heavier bulbs than native and invasive sexual plants (*P <* 0.05), while obligated asexuals had fairly heavy, but highly heterogeneous bulbs that did not differ significantly from the other invasive groups (**Figure 2E**). There was a clear and significantly higher investment in total bulb weight by the invasive sexual and predominantly asexual plants than the other groups, as well as in the obligated asexual individuals in comparison with native sexual plants (*P <* 0.05; **Figure 2F**).

### Trade-off between Sexual and Asexual Investment

**FIGURE 3:**
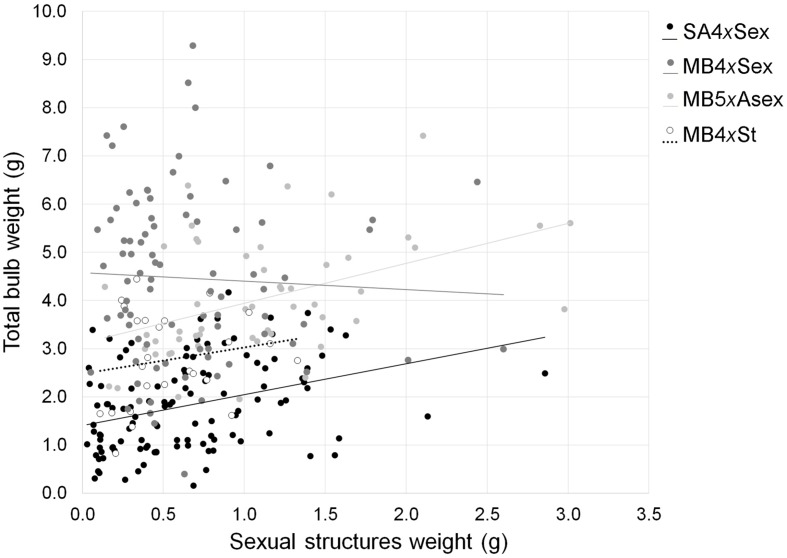
**Correlation between biomass invested in the production of sexual and asexual structures among different forms of *Oxalis pes-caprae* from its native and invaded areas and with distinct reproductive strategies: South African 4*x* sexual forms (SA4*x*Sex), Mediterranean basin 4*x* sexual forms (MB4*x*Sex), Mediterranean basin 5*x* predominantly asexual form (MB5*x*Asex) and Mediterranean basin 4*x* obligated asexual form (MB4*x*St, sterile double-flowered form)**.

No trade-off was observed in the biomass invested in sexual and asexual structures. On the contrary, the production of sexual structures was positively correlated with the biomass invested in the production of bulbs, except for invasive sexuals and obligated asexuals (total: *r* = 0.214, *P <* 0.001; analyses by group: SA4*x*Sex: *r* = 0.286, *P* = 0.0182; MB4*x*Sex: *r* = -0.0223, *P* = 0.800; MB5*x*Asex: *r* = 0.449, *P <* 0.001; MB4*x*St: *r* = 0.183, *P* = 0.381; **Figure 3**).

## Discussion

Our results indicate the occurrence of evolutionary changes in the reproductive traits of invasive populations of *O. pes-caprae*. Indeed, most of the traits evaluated, differed between native and invasive populations and among individuals with different reproductive strategies when grown in the same environment. In particular, we found that: (1) overall, plants with sexual ability (including the 5*x* S-morph) had higher probability of flowering and larger floral displays than the sterile forms; (2) the total investment in the production of floral structures was significantly higher in the 5*x* form than in the remaining forms; this suggests an effect of the ploidy level in the overall size of the structures and, in the case of the sterile double-flowered form, a trade-off between the number of flowering structures and the resources needed to produce them (i.e., heavier sterile flowers resulting in lower number of inflorescences); (3) differences in the production of bulbs and seeds revealed that native plants had higher sexual fitness, while a transition toward clonality was clear for the invasive forms; (4) differences were also observed among invasive individuals, with the sexual forms producing more dispersal units (seeds and small bulbs), the predominantly asexual form producing an inter-medium number of large bulbs, and the sterile form being apparently less aggressive and producing less, yet large, bulbs; (5) finally, no trade-off between sexual and asexual investments was observed. Below, we discuss our results in light of the complex invasion history of *O. pes-caprae* and draw hypotheses on how reproductive traits could have been involved in the invasion success and in the prevalence of some forms in the invaded range of the western Mediterranean region.

### Reproduction: Traits and Strategies

Reproduction determines the number and genetic composition of dispersal units, being vital for the establishment and spread of plant populations after long-distance dispersal (e.g., Sakai et al., 2001; Ness et al., 2010). The relative contribution of different reproductive modes varies depending on the ecological and genetic factors under which colonizers are subjected (e.g., Dorken and Eckert, 2001; Eckert, 2002; Herben et al., 2015). Our results showed remarkable differences in several reproductive traits between ranges and among forms with different reproductive strategies. Overall, a transition to clonality was observed among invasive plants: native individuals had a higher sexual fitness than invasive ones, which in contrast had higher asexual fitness than natives. These observations matched our expectations mainly by two reasons, described below.

Firstly, genetic diversity of native populations is higher than that of invasive ones (Ferrero et al., 2015), and this is expected to affect the sexual fitness. Sexual reproduction is the main mechanism of reproduction in the native range, where floral polymorphism promotes outcrossing and thus genetic diversity and frequency dependent selection governs isoplethic populations (Ornduff, 1987; Turketti, 2010; Ferrero et al., 2015). Contrarily, invasive populations are highly constrained by the scarcity of compatible mates (Castro et al., 2007, 2013), being dominated by 5*x* S-morphs with residual sexual reproduction (Costa et al., 2014, in press), and thus with low recombination probabilities. Additionally, strong founder effects led to a decrease in genetic diversity of invasive populations (Ferrero et al., 2015). Altogether, these factors significantly impacted genetic composition of invasive populations and, consequently, plant fitness under outcross pollinations. Genetic depauperation after long distance dispersal has been described in several other invasive species (e.g., Dlugosch and Parker, 2008; Zhang et al., 2010), and although multiple introductions can ameliorate their consequences (Novak and Mack, 2005; Dlugosch and Parker, 2008; Simberloff, 2009), negative impacts of low genetic diversity for plant reproduction have been shown (Barrett, 2002; Crawford and Whitney, 2010). However, genetic bottlenecks do not necessarily hinder the adaptive potential of invasive species (Barrett et al., 2008; Dlugosch and Parker, 2008; Rollins et al., 2013).

Secondly, under unfavorable conditions for sexual reproduction in invasive populations, i.e., strong mate limitation (either due to monomorphic populations or due to the predominance of asexual 5*x* individuals; Baker, 1965; Ornduff, 1987; Castro et al., 2007, 2013; Ferrero et al., 2015), we expected that selection would benefit individuals with an increased capacity for investment in asexual reproduction. Indeed, uniparental reproduction has been proposed to be selectively advantageous under scenarios of strong mate limitation, such as invasions, rapid range expansion, island colonization and meta-population dynamics (Baker’s Law; Baker, 1965; Pannell et al., 2015). Our results corroborate this prediction: regardless of the reproductive strategy, invasive *O. pes-caprae* forms invested significantly more in asexual reproduction than natives. An enhancement in clonal reproduction in invasive populations in comparison with natives has also been observed in several other species. For example, invasive *Butomus umbellatus* individuals were more likely to produce bulbils than native individuals (Brown and Eckert, 2005); rapid selection of genotypes with stronger vegetative growth was observed in *Phalaris arundinacea* (Lavergne and Molofsky, 2007), and greater vegetative reproduction in the invasive ranges of *Achillea millefolium* and *Hypericum perforatum* than on their native ranges (Beckmann et al., 2009). Interestingly, differences in bulb production have also been observed among invasive populations of *O. pes-caprae* in the Mediterranean basin, with insular populations having higher dispersal potential than continental ones (Vilà and Gimeno, 2006). Clonal reproduction was one of the traits identified by Baker (1965) for the “ideal weed” and there are several studies addressing invasive species traits that support this reproductive strategy as one of the features involved with successful invasions (e.g., Pyšek and Richardson, 2007; Silvertown, 2008; Marco et al., 2010).

As expected, individuals with the ability to reproduce sexually, including the predominantly asexual plants, invested more in the production of floral structures than the sterile form. This is in accordance with a strategy to promote sexual reproduction either by investing in attractive floral display (i.e., larger inflorescences, inflorescences with larger flower displays) or by producing more sexual potential units (Barrett, 2002). A trade-off between the number of inflorescences produced and the considerable amount of energy necessary to produce double-flowers might explain the reduced floral display of the sterile double-flowered form (see below). Interestingly, the 5*x* form produced larger flowers and larger inflorescences that resulted in a higher biomass investment without a detrimental impact in the floral display. The production of larger reproductive structures is likely related with the ploidy level, since polyploidy is hypothesized to drive significant changes in cell size and, consequently, in overall organ size (Levin, 2002). Apparently, this higher investment did not lead to allocation trade-offs since the 5*x* cytotype produced similar floral display to other sexual forms.

Besides the differences in the production of sexual and asexual diaspores detected between ranges, different strategies were also observed among invasive forms. As expected, sexual fitness was higher in the 4*x* sexual forms than in the 5*x* form, and null in the sterile double-flowered form where the sexual organs were replaced by petals due to a mutation in the genes responsible for the floral development (Weigel and Meyerowitz, 1994). The lower sexual fitness of the 5*x* individuals after outcrossing is mainly due to its odd ploidy level; although these 5*x* individuals are able to produce some viable gametes, they also produce unviable gametes with variable ploidies (Vignoli, 1937; Signorini et al., 2013; Costa et al., 2014), diminishing significantly the production of offspring through seed. However, and as described above, the 5*x* individuals produced slightly larger floral displays and bigger floral structures; these features increase the number of gametes and the attractiveness of the plants for pollinators, which might contribute to ameliorate the low sexual potential of the 5*x* individuals. Additionally, differences in the asexual traits were also detected among invasive forms. Under optimal resource conditions, the obligated asexuals invested significantly less in bulb production than the other invasive forms, producing larger bulbs but in smaller amounts (like native plants) than the other invasive forms. Interestingly, the sexual and predominately asexual forms allocated a similar amount of energy to the production of bulbs; however, while the former invested resources in producing many small bulbs, the later invested in less but larger bulbs. These patterns agree with trade-off models for propagule number and size which predict that in optimal environments it is preferable to maximize offspring quantity, whereas in stressful conditions (such as the limitation of sexual partners) it is preferable to invest in offspring quality (Smith and Fretwell, 1974; Sadras, 2007). Based on this model, we could hypothesize that selective pressures during the invasion of the predominantly asexual form might have benefited larger bulbs, while selective pressures over asexual propagule production are not expected to be so strong for the sexual forms that have an additional reproductive mode (Costa et al., unpublished results). *Oxalis pes-caprae* bulb weight has been pointed as an important feature of the invasion process, especially under stressful conditions (Lane, 1984; Sala et al., 2007), with parental bulb weight significantly impacting plant biomass in shaded environments, as evident by the production of significantly more leaves in plants originated from bigger bulbs than from smaller ones (Verdaguer et al., 2010). However, although fitting nicely the results, there are several lines of evidence that do not completely support this hypothesis and make our findings difficult to interpret. First, it is difficult to disentangle the effects of ploidy level from those related with evolutionary changes. Although, bulb size of the offspring of 4*x* sterile double-flowered form and the 5*x* S-morph were similar, the larger bulbs in the 5*x* individuals might be driven by ploidy, similarly to the pattern observed in the flowering structures (results herein) and to the patterns observed in other polyploid complexes (Levin, 2002). Second, 5*x* S-morph individuals showed that parent bulb weight has a small overall effect on *O. pes-caprae* plant biomass (Sala et al., 2007; Verdaguer et al., 2010). In general, bulbs emerged successfully and vigorously regardless of their sizes (Vilà et al., 2006; Verdaguer et al., 2010), still, parent bulb size might be particularly important for plant emergence and initial development, depending on the conditions where the plant is growing (Vilà et al., 2006; Sala et al., 2007; Verdaguer et al., 2010). Regardless of the effects in early stages, bulb size was not determinant for the development of adult plants and subsequent offspring production, possibly because further plant growth might become independent of this storage organ once the plant starts to photosynthesize. Finally, the production of bulbs in *O. pes-caprae* was shown to be plastic and highly dependent of nutrient availability (Sala et al., 2007). If bulb weight has no fitness advantage, then producing many small bulbs would be advantageous, especially when mate limitation is strong and no allocation trade-off between sexual and asexual investment is observed (see below). In this context, sexual invasive forms have a higher dispersal potential, through both sexual and asexual means than the other invasive forms and might become widespread in the future.

### Bermuda Buttercup Invasion History: What Have We Learned So Far?

The Bermuda buttercup is a classic example in biological invasions, known as a strictly asexual form that successfully spread in Mediterranean climate regions of the world (5*x* S-morph; Baker, 1965). However, the origin of this invasive form is still unclear and the colonization history revealed to be more complex and dynamic than previously envisaged. Native populations are composed of the 4*x* cytotype, with the 5*x* S-morph being extremely rare (Michael, 1964; te Beest et al., 2012; Signorini et al., 2013; Ferrero et al., 2015). Contrarily, the 5*x* S-morph dominates all Mediterranean climate regions, except in Australia where both asexual (monomorphic 5*x* S-morph populations) and sexual populations (4*x* trimorphic populations) have been reported (Symon, 1961; Michael, 1964; Ferrero, personal observations). The most accepted hypothesis is that the 5*x* S-morph has been originated from 4*x* individuals in the introduced range and subsequently introduced in several areas of the world (Krejčíková et al., 2013; Signorini et al., 2013; Ferrero et al., 2015; most probably multiple times in the Mediterranean basin, Signorini et al., 2011), including South Africa, where it was recently reported in a new semi-natural location for the first time (Ferrero et al., 2015). The combination of several factors, including strong heteromorphic incompatibility system, lack of compatible mates and odd ploidy, constrained the production of dispersal units mostly to asexual means, and consequently the successful spread of this form in introduced ranges became dependent on bulb production (Baker, 1965; Ornduff, 1987). Our results strongly support this hypothesis showing a clear selection toward clonality through a significantly increase in the number of bulbs as well as in their size (the latter driven or not exclusively by the ploidy level) in comparison with natives. Producing more bulbs would be selectively advantageous since it increases the number of propagules, while larger bulbs may confer significant advantages under stressful environments, allowing faster plant emergence and providing more reserves, which will translate into larger plants (Vilà and Gimeno, 2006; Sala et al., 2007; Verdaguer et al., 2010; Tavares, 2014).

However, the story does not end here. In the western Mediterranean region, invasive populations seem to be changing very rapidly (Castro et al., 2007, 2013; Costa et al., 2014, in press). Molecular studies have shown an invasion punctuated by multiple introductions of other floral morphs comprised of the tetraploid ploidy level (Ferrero et al., 2015), and field surveys detected a reacquisition of sexual reproduction in this region (Castro et al., 2013; Costa et al., 2014, in press). Although the introduction or incipient occurrence of M- and L-morphs would constitute a source of compatible mates, these individuals are still under a scenario of strong mate limitation due to the dominance of the predominantly asexual 5*x* S-morph. Thus, they will be subjected to similar strong selective pressures toward uniparental reproduction. Our results clearly support that these new individuals have also diverged from native populations and present an inversion toward uniparental reproduction via asexual reproduction (results herein), but also via changes in the strength of the incompatibility system (Costa et al., unpublished results). Additionally, our results show that these sexual forms have superior reproductive fitness in comparison with the 5*x* S-morph and the 4*x* sterile double-flowered form. So, how can we explain the distribution patterns in the western Mediterranean basin? Based on the reproductive traits, the current distribution patterns can only be explained under a scenario of different introduction timings, first with the introduction and spread of the 5*x* individuals and more recently with the introduction of 4*x* sexual plants that are starting to become more dominant than previously documented. Given the superior reproductive fitness of the 4*x* sexual individuals, they are expected to become more dominant in the future. Still, other life-history transition stages and ecological responses, including bulb viability and emergence, competitive ability, resistance to herbivory and response to soil disturbance, need to be addressed in future studies in order to fully characterize the fitness of each form.

The successful spread of the sterile double-flowered form in south–west Iberian Peninsula is particularly intriguing. This form had the lowest dispersal potential among invasive forms and therefore it is likely under a competitive disadvantage with other floral forms. Recent molecular studies have shown a close relationship between these individuals and native plants, supporting the occurrence of several multiple introductions. These multiple introductions might have provided a sufficiently high number of propagules to mediate a successful invasion process. Although species traits are extremely important, several studies have shown that propagule pressure is also a determinant factor for successful invasion (Novak and Mack, 2005; Colautti et al., 2006; Dlugosch and Parker, 2008; Simberloff, 2009). Interestingly, propagule pressure was also shown to be important in colonization by *O. pes-caprae* along altitudinal gradients within invaded areas (Ross et al., 2008). Additionally, besides ecological and life history traits, human mediated dispersal (e.g., in earlier stages as ornamental plant, and currently through soil movements in agriculture, horticulture and gardening, or through land translocations during road constructions; Michael, 1964; Signorini et al., 2011; Castro et al., 2013) might have also promoted the dispersal of this invasive form, as well as the others (Pyšek and Richardson, 2007).

### Trade-offs between Sexual and Asexual Strategies

No trade-off between the production of sexual and asexual structures has been detected in *O. pes-caprae*. These observations agree with studies in other species (Vallejo-Marín et al., 2010; Van Drunen and Dorken, 2012) and with previous experiments with *O. pes-caprae* (Vilà and Gimeno, 2006; Verdaguer et al., 2010). This lack of a trade-off might be explained by the particular developmental processes of the plant as the production of flowering structures and bulbs are asynchronous in *O. pes-caprae* likely reducing the competition for resources between both reproductive processes. In the first half of the plants’ life cycle, most of the energy is redirected to growth and flowering, and only afterward, when the aboveground part of the plant starts to senesce, energy is directed to the production of underground structures, namely to the production of bulbs (Pütz, 1994; Verdaguer et al., 2010). Indeed, we observed the opposed pattern, with a positive correlation between bulb and flower biomass. This could simply be a reflection of plant size rather than resource management strategies.

## Conclusion

Different sexual and asexual reproductive traits were quantified between native and invasive populations, as well as among different forms within invasive populations. Different reproductive strategies and ecological and genetic contexts created by long-distance dispersal seem to generate divergent selective pressures in several sexual and asexual reproductive traits. The introduction process seems to have promoted clonal reproduction and this is most probably the major trait driving the invasion success of *O. pes-caprae*; however, invasive sexual forms have increased dispersal potential and additional means to produce dispersal units and promote heterozygosity. Consequently, invasive sexual forms are expected to be in competitive advantage in relation to the predominately asexual and obligated asexual plants, and thus could become widespread in the invaded range in the future. Historical processes, with the introduction of the predominantly asexual 5*x* S-morph first and more recently of the 4*x* sexual morphs, were probably important in establishing the current distributional patterns of the different forms in the western Mediterranean basin. This study shows that invasion processes can be incredibly complex and dynamic, while the interaction between ecological and genetic constraints determined by the invasion process might result in different reproductive strategies which in turn determine the success of invasive populations.

## Author Contributions

SC, VF, LN, and JL designed the experiment; SC, VF, JC, and JL conducted field collections; MC, with the collaboration of all the authors, conducted the common garden experiment; SC analyzed the data with the other authors participating in the discussion of the results; SC and MC, with contribution of all the authors, wrote the manuscript.

## Conflict of Interest Statement

The authors declare that the research was conducted in the absence of any commercial or financial relationships that could be construed as a potential conflict of interest.
