# Supplementary material for: Invasion Fosters Change: Independent Evolutionary Shifts in Reproductive Traits after Oxalis pes-caprae L. Introduction
Source: Front Plant Sci. 2016 Jun 24;7:874. doi: 10.3389/fpls.2016.00874 (PMC4919335; doi:10.3389/fpls.2016.00874)
Supplement: Supplementary file 1 [file Table_1.DOC]

**Invasion fosters the change: independent evolutionary shifts in reproductive traits after *Oxalis pes-caprae* L. introduction**

Sílvia Castro*, Mariana Castro, Victoria Ferrero, Joana Costa, Daniela Tavares, Luis Navarro, João Loureiro

*Correspondence: Sílvia Castro: [**scastro@bot.uc.pt**](mailto:scastro@bot.uc.pt)

Supplementary Table 1. *Oxalis pes-caprae* populations from the native range of South Africa and invaded range of the Western Mediterranean basin used to study sexual and asexual reproductive traits

| **Population** | **Geographical coordinates** | | **n** | **Floral forms (%)** | | | |
| --- | --- | --- | --- | --- | --- | --- | --- |
|  |  |  |  | **St** | **L-** | **M-** | **S-** |
| **Native range: South Africa** | | | **137** |  |  |  |  |
| ZA: Yzerfontein | 33º20.979'S | 18º09.302'E | 7 | 0 | 32 | 22 | 46 |
| ZA: Langebaan | 33º03.495'S | 18º04.716'E | 2 | 0 | 58 | 17 | 25 |
| ZA: Dwarskersbos | 32º36.558'S | 18º19.059'E | 2 | 0 | 45 | 37 | 18 |
| ZA: Lamberts Bay | 32º11.517'S | 18º19.924'E | 7 | 0 | 28 | 26 | 46 |
| ZA: Clanwilliam | 32º7.526'S | 18º51.857'E | 1 | 0 | 52 | 23 | 25 |
| ZA: Klawer | 31º45.478'S | 18º34.855'E | 3 | 0 | 33 | 43 | 24 |
| ZA: Doringbos | 32º06.999'S | 19º03.087'E | 4 | 0 | 41 | 41 | 18 |
| ZA: Citrusdal | 32º19.006'S | 18º54.726'E | 1 | 0 | 39 | 32 | 29 |
| ZA: Porteville | 32º44.012'S | 18º54.596'E | 4 | 0 | 38 | 34 | 28 |
| ZA: Gouda | 32º13.192'S | 18º58.442'E | 3 | 0 | 23 | 29 | 48 |
| ZA: Riebbek Wes | 33º13.221'S | 18º43.259'E | 3 | 0 | 21 | 42 | 38 |
| ZA: Paarl | 33º41.074'S | 18º45.868'E | 3 | 0 | 8 | 73 | 20 |
| ZA: Worcester | 33º33.671'S | 19º54.072'E | 5 | 0 | 40 | 37 | 22 |
| ZA: Robertson | 33º48.877'S | 18º54.072'E | 3 | 0 | 30 | 34 | 36 |
| ZA: Suurbraak | 34º3.469'S | 20º35.526'E | 5 | 0 | 53 | 16 | 32 |
| ZA: Riversdal | 34º4.648'S | 21º14.651'E | 2 | 0 | 65 | 14 | 22 |
| ZA: Barrydale | 33º47.247'S | 21º08.652'E | 7 | 0 | 37 | 23 | 40 |
| ZA: Oudtshoorn | 33º32.827'S | 21º50.612'E | 1 | 0 | 39 | 56 | 5 |
| ZA: Mossel Bay | 34º5.665'S | 22º03.400'E | 2 | 0 | 20 | 51 | 29 |
| ZA: Gouritsmond | 34º17.703'S | 21º49.356'E | 4 | 0 | 30 | 38 | 32 |
| ZA: Stilbaai | 34º21.241'S | 21º25.003'E | 4 | 0 | 21 | 41 | 38 |
| ZA: Witsand | 34º15.118'S | 20º59.556'E | 8 | 0 | 24 | 41 | 35 |
| ZA: Bredasdorp | 34º18.129'S | 20º12.213'E | 8 | 0 | 33 | 21 | 46 |
| ZA: L'Agulhas | 34º41.391'S | 20º1.198'E | 8 | 0 | 24 | 24 | 52 |
| ZA: Elim | 34º35.961'S | 19º45.557'E | 4 | 0 | 1 | 78 | 21 |
| ZA: Standford | 34º27.371'S | 19º35.046'E | 6 | 0 | 36 | 3 | 60 |
| ZA: Caledon | 34º10.961'S | 19º24.159'E | 13 | 0 | 8 | 11 | 81 |
| ZA: Botrivier | 34º13.400'S | 19º11.994'E | 7 | 0 | 26 | 70 | 4 |
| ZA: Cape Point | 34º09.413'S | 18º26.100'E | 10 | 0 | 15 | 76 | 8 |
| **Invaded range: Western Mediterranean basin** | | | **201** |  |  |  |  |
| SP: Baiona | 42° 6.747'N | 8° 49.636'W | 6 | 0 | 0 | 0 | 100 |
| PT: Praia de Mira | 40° 27.257'N | 8° 46.756'W | 3 | 0 | 0 | 0 | 100 |
| PT: Coimbra | 40°12.363'N | 8°25.431'W | 4 | 0 | 0 | 0 | 100 |
| PT: Colares III | 38º48.752'N | 9º28.394'W | 24 | 0 | 39 | 13 | 48 |
| PT: Colares I | 38º48.015'N | 9º28.061'W | 42 | 0 | 50 | 22 | 28 |
| PT: Colares II | 38° 47.863'N | 9° 28.577'W | 14 | 0 | 63 | 18 | 19 |
| PT: Colares IV | 38° 48.274'N | 9° 28.288'W | 31 | 0 | 36 | 6 | 58 |
| PT: Troia | 38°29.495'N | 8°54.386'W | 9 | 0 | 5 | 0 | 95 |
| PT: Melides | 38°07.843'N | 8°46.961'W | 16 | 31 | 0 | 0 | 69 |
| PT: Almograve | 37°38.885'N | 8°47.320'W | 26 | 9 | 0 | 0 | 90 |
| PT: Armação de Pêra | 37°04.856'N | 8°17.201'W | 11 | 19 | 0 | 0 | 81 |
| MA: Moulay-Bousselham | 34°52.542'N | 6°17.831'W | 12 | 0 | 31 | 0 | 69 |
| MA: Essaouira | 31° 29.722'N | 9° 45.638'W | 3 | 0 | 4 | 0 | 96 |

Floral forms: St, sterile double-flowered form; L-, M- and S- for long-styled, mid-styled and short-styled morphs, respectively. Countries: SP, Spain; PT, Portugal; MA, Morocco; ZA, South Africa. Each population is characterized for the frequency of the floral forms where given in percentage.
