# Supplementary material for: Invasion Fosters Change: Independent Evolutionary Shifts in Reproductive Traits after Oxalis pes-caprae L. Introduction
Source: Front Plant Sci. 2016 Jun 24;7:874. doi: 10.3389/fpls.2016.00874 (PMC4919335; doi:10.3389/fpls.2016.00874)
Supplement: Supplementary file 2 [file Table_2.DOC]

**Invasion fosters the change: independent evolutionary shifts in reproductive traits after *Oxalis pes-caprae* L. introduction**

Sílvia Castro*, Mariana Castro, Victoria Ferrero, Joana Costa, Daniela Tavares, Luis Navarro, João Loureiro

*Correspondence: Sílvia Castro: [**scastro@bot.uc.pt**](mailto:scastro@bot.uc.pt)

Supplementary Table 2. Results of the statistical analyses performed in this study. Reproductive strategy and area combined were defined as fixed factor to assess differences in sexual and asexual traits (response variables), with initial bulb weight included as covariate; individual and population defined as random factors [(1|ind) and (1|pop), respectively] in generalized linear mixed models (GLMM); the random factors were removed from the analyses when their variance was lower than the variance of the residuals and a generalized linear model (GLM) was used instead

|  |  | **Fixed factor:** reproductive strategy and area combined | | |  | **Covariate:** Initial bulb weight | | |  | **Model** |
| --- | --- | --- | --- | --- | --- | --- | --- | --- | --- | --- |
| **Response variables** | **n** | **d.f** | ***χ^2^*** | ***P*** |  | **d.f.** | ***χ^2^*** | ***P*** |  |
| Bulb viability | 338 | 3 | 3.49 | 0.322 |  | 1 | **8.61** | **0.003** |  | GLM |
| Probability of flowering | 305 | 3 | **11.23** | **0.011** |  | 1 | 1.25 | 0.264 |  | GLM |
| No. of flowers per inflorescence | 276 | 3 | **19.07** | **<0.001** |  | 1 | 2.44 | 0.119 |  | GLM |
| No. inflorescences per plant | 276 | 3 | **47.89** | **<0.001** |  | 1 | 1.06 | 0.303 |  | GLM |
| Total no. flowers per plant | 276 | 3 | **31.68** | **<0.001** |  | 1 | 0.20 | 0.658 |  | GLMM: (1|ind), (1|pop) |
| Mean flower weight | 276 | 3 | **820.80** | **<0.001** |  | 1 | 0.35 | 0.556 |  | GLMM: (1|ind) |
| Mean inflorescence weight | 276 | 3 | **51.78** | **<0.001** |  | 1 | 0.01 | 0.925 |  | GLMM: (1|ind) |
| Total flower weight | 276 | 3 | **28.08** | **<0.001** |  | 1 | 0.20 | 0.654 |  | GLMM: (1|ind), (1|pop) |
| Total inflorescence weight | 276 | 3 | **44.39** | **<0.001** |  | 1 | **282.83** | **<0.001** |  | GLMM: (1|ind) |
| Total weigh flowering structures | 276 | 3 | **32.30** | **<0.001** |  | 1 | 1.03 | 0.311 |  | GLMM: (1|ind), (1|pop) |
| Fruit set | 251 | 2 | **47.38** | **<0.001** |  | 1 | 0.00 | 0.999 |  | GLMM: (1|ind), (1|pop) |
| Seed set | 251 | 2 | **89.44** | **<0.001** |  | 1 | 2.20 | 0.138 |  | GLMM: (1|ind), (1|pop) |
| Sexual potential per plant | 251 | 2 | **15.77** | **<0.001** |  | 1 | **110.02** | **<0.001** |  | GLMM: (1|ind), (1|pop) |
| No. of bulbs per plant | 305 | 3 | **350.13** | **<0.001** |  | 1 | **6.40** | **0.011** |  | GLM |
| Mean bulb weight | 305 | 3 | **27.36** | **<0.001** |  | 1 | 0.24 | 0.621 |  | GLM |
| Total bulb weight | 305 | 3 | **211.13** | **<0.001** |  | 1 | 0.35 | 0.554 |  | GLM |
