# Supplementary material for: Invasion Fosters Change: Independent Evolutionary Shifts in Reproductive Traits after Oxalis pes-caprae L. Introduction
Source: Front Plant Sci. 2016 Jun 24;7:874. doi: 10.3389/fpls.2016.00874 (PMC4919335; doi:10.3389/fpls.2016.00874)
Supplement: Supplementary file 3 [file Data_Sheet_1.DOC]

**Invasion fosters the change: independent evolutionary shifts in reproductive traits after *Oxalis pes-caprae* L. introduction**

Sílvia Castro*, Mariana Castro, Victoria Ferrero, Joana Costa, Daniela Tavares, Luis Navarro, João Loureiro

*Correspondence: Sílvia Castro: [**scastro@bot.uc.pt**](mailto:scastro@bot.uc.pt)


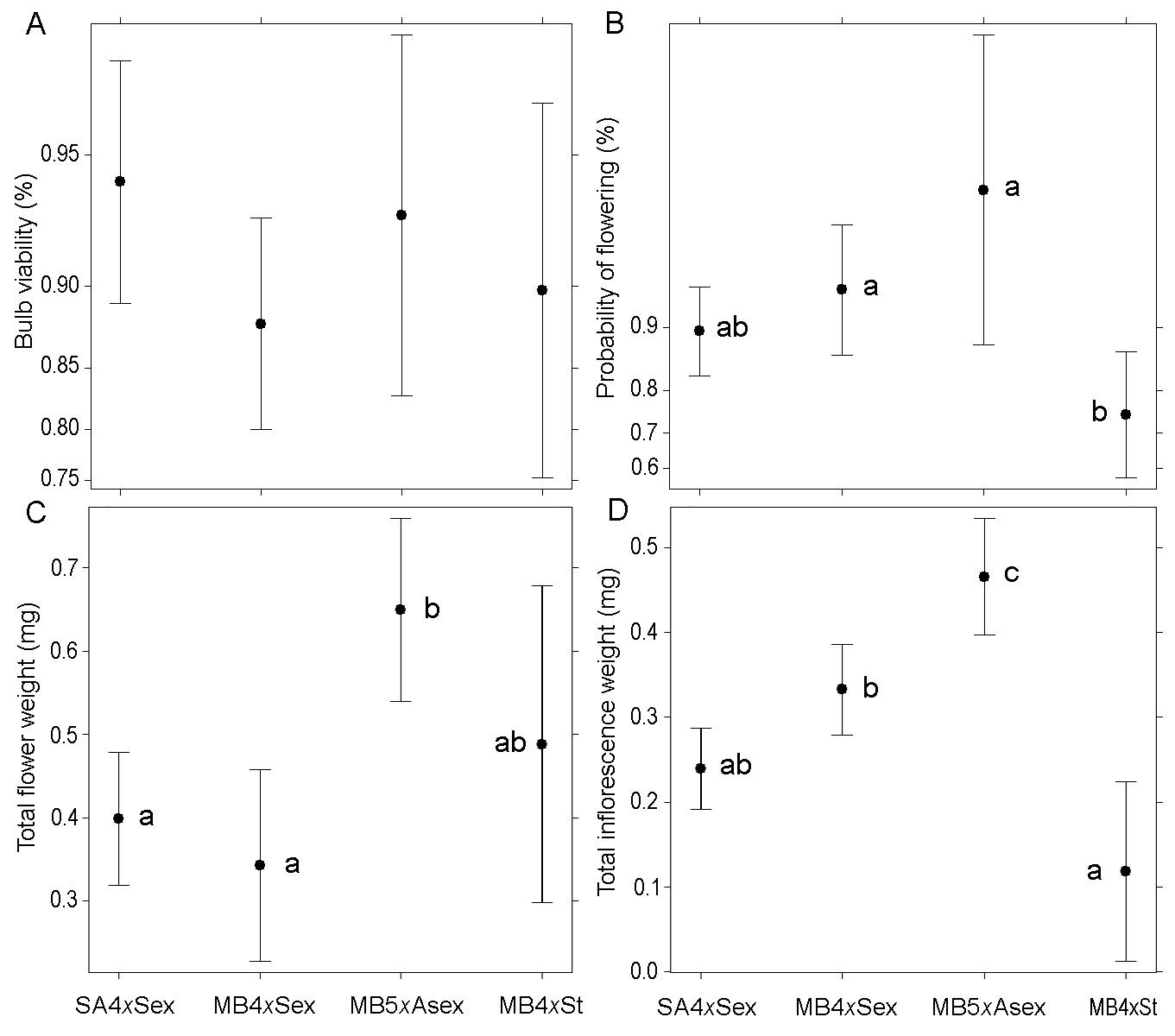
**Supplementary Figure 1. Additional traits assessed among different forms from native and invaded areas with distinct reproductive strategies.** South African 4*x* sexual forms (SA4*x*Sex), Mediterranean basin 4*x* sexual forms (MB4*x*Sex), Mediterranean basin 5*x* predominantly asexual form (MB5*x*Asex) and Mediterranean basin 4x obligated asexual form (MB4*x*St, sterile double-flowered form). (**A**) Bulb viability, given as the proportion of bulbs that have originate a plant; (**B**) Flowering, given as a probability of a plant to produce flowering structures; (**C**) Total weight of the flowers produced per plant (mg); (**D**) Total weight of the inflorescences produced per plant (mg). Values are given as model-adjusted back-transformed least-square means and 95% confident intervals. Significant differences among factors are indicated with different letters (*P <* 0.05).
